# Interferon-Induced Transmembrane Protein (IFITM3) Is Upregulated Explicitly in SARS-CoV-2 Infected Lung Epithelial Cells

**DOI:** 10.3389/fimmu.2020.01372

**Published:** 2020-06-10

**Authors:** Mahmood Yaseen Hachim, Saba Al Heialy, Ibrahim Yaseen Hachim, Rabih Halwani, Abiola C. Senok, Azzam A. Maghazachi, Qutayba Hamid

**Affiliations:** ^1^Sharjah Institute for Medical Research, College of Medicine, University of Sharjah, Sharjah, United Arab Emirates; ^2^College of Medicine, Mohammed Bin Rashid University of Medicine and Health Sciences, Dubai, United Arab Emirates; ^3^Meakins-Christie Laboratories, Research Institute of the McGill University Health Center, Montreal, QC, Canada

**Keywords:** COVID-19, SARS-CoV-2, interferon-induced transmembrane proteins, valproic acid, antiviral immunity

## Abstract

Current guidelines for COVID-19 management recommend the utilization of various repurposed drugs. Despite ongoing research toward the development of a vaccine against SARS-CoV-2, such a vaccine will not be available in time to contribute to the containment of the ongoing pandemic. Therefore, there is an urgent need to develop a framework for the rapid identification of novel targets for diagnostic and therapeutic interventions. We analyzed publicly available transcriptomic datasets of SARS-CoV infected humans and mammals to identify consistent differentially expressed genes then validated in SARS-CoV-2 infected epithelial cells transcriptomic datasets. Comprehensive toxicogenomic analysis of the identified genes to identify possible interactions with clinically proven drugs was carried out. We identified IFITM3 as an early upregulated gene, and valproic acid was found to enhance its mRNA expression as well as induce its antiviral action. These findings indicate that analysis of publicly available transcriptomic and toxicogenomic data represents a rapid approach for the identification of novel targets and molecules that can modify the action of such targets during the early phases of emerging infections like COVID-19.

## Introduction

Coronaviruses are a large family of viruses that were first described over 50 years ago (1). Since the turn of the millennium, there have been two major global outbreaks caused by coronaviruses, namely SARS-CoV in 2003 and MERS-CoV in 2012 (2). The ongoing COVID-19 pandemic caused by SARS-CoV-2 represents the third and most devastating of these outbreaks. These outbreaks, notably the COVID-19 pandemic, are harsh reminders of the challenges posed by emerging infectious diseases. The global impact of the COVID-19 pandemic has brought to the forefront the need to rapidly develop and deploy an effective vaccine. However, despite ongoing concerted research efforts, it is accepted that such a vaccine will not be available in time to contribute to the containment of the ongoing pandemic. Current management guidelines include the use of repurposed drugs such as chloroquine and its analog hydroxychloroquine as well as antiviral agents (3). However, the need for well-designed clinical trials to validate their efficacy continues to be highlighted. To effectively address the ongoing COVID-19 pandemic, there is a recognized need for a framework for rapid identification of novel targets for diagnostic and therapeutic interventions as well as determine clinically approved drugs with high potential for repurposed use against SARS-CoV-2. Publicly available transcriptomic datasets generated from SARS-CoV infected humans, and mammalian cells represent a wealth of data that could be used to identify consistent differentially expressed genes, which could then be validated against SARS-CoV-2 infected epithelial cells transcriptomic datasets. A comprehensive toxicogenomic analysis of the identified genes could potentially identify possible interactions with clinically proven drugs. This simple approach can be used for the rapid identification of novel targets and drugs for further validation. In this study, we have applied this approach, and our findings have identified IFITM3 as an early upregulated gene and indicate that valproic acid enhances IFITM3 mRNA expression and antiviral action.

## Materials and Methods

Publicly available transcriptomic datasets were retrieved from Gene Expression Omnibus (GEO) (https://www.ncbi.nlm.nih.gov/geo/). Only microarray gene expression datasets with the word “SARS-COV,” virus, or modified strain infected vs. mock-infected and no more than 48 h after the infection. Twelve datasets fulfilled the criteria, as detailed in Table 1. We used GEOquery and limma R packages through the GEO2R tool for each dataset (12). After sorting the genes according to the False Discovery Rate (FDR), the top 2,000 differentially expressed probes with FDR <0.05 were selected from each dataset. The annotated genes of the 5,000 probes in each dataset were intersected with differentially expressed genes (DEGs) from all other datasets. The DEGs that were common in at least 9 out of the 12 (75%) datasets were identified as shared genes that are consistently DEG in the first 48 h of SARS-COV infection. Enriched Ontology Clustering for the identified genes was performed to explore using the Metascape (http://metascape.org/gp/index.html#/main/step1). The shortlisted genes expression was then explored in another dataset (GSE147507), where RNA-Sequencing of primary human lung epithelium (NHBE) mock-treated or infected with SARS-CoV-2 was done to examine whether there is a difference in the response of SARS-CoV-2 from other strains in terms of DEGs (13).

**Table 1:** List of publicly available transcriptomics datasets retrieved from Gene Expression Omnibus (GEO) and used in the study.

**No**.	**Study title**	**Model**	**Strain**	**Gene set ID**	**References**
1.	Absence of host innate immune responses in SARS-CoV-infected ferrets upon subsequent challenge	Ferret	SARS-CoV (TOR2)	GSE11704	(4)
2.	Dynamic innate immune responses of human bronchial epithelial cells against SARS-CoV and DOHV infection	2B4 cells, a clonal derivative of Calu-3 cells	Urbani strain of SARS-CoV	GSE17400	(5)
3.	Comparative pathogenesis of three human and zoonotic SARS-CoV strains in cynomolgus macaques	Cynomolgus macaques	Recombinant SARS-CoV bearing variant S glycoproteins (Urbani, GZ02 and HC/SZ/6103)	GSE23955	(6)
4.	SM001: SARS CoV MA15 infection of C57Bl/6 mouse model – data from 4 viral doses at 1, 2, 4 and 7 days post infection.	C57BL/6 mice	SARS CoV MA15	GSE33266	(7)
5.	SCL005: icSARS CoV Urbani or icSARS deltaORF6 infections of the 2B4 clonal derivative of Calu-3 cells - Time course	Calu-3 cells	icSARS CoV or the icSARS deltaORF6 mutant	GSE33267	(8)
6.	SCL006,icSARS CoV urbani or icSARS Bat SRBD (spike receptor binding domain from the wild type strain urbani to allow for infection of human and non-human primate cells) infections of the 2B4 clonal derivative of calu-3 cells - Time course	Calu-3 cells	icSARS CoV or the cSARS Bat SRBD strain	GSE37827	(8)
7.	SHAE002: SARS-CoV, SARS-dORF6 and SARS-BatSRBD infection of HAE cultures.	HAE cultures	SARS-CoV, SARS-dORF6 or SARS-BatSRBD	GSE47960	(9)
8.	SCL008: icSARS CoV, icSARS-deltaNSP16 or icSARS ExoNI infections of the 2B4 clonal derivative of Calu-3 cells - Time course	2B-4 cells (clonal derivatives of Calu-3 cells)	icSARS CoV, icSARS deltaNSP16 or icSARS ExoNI	GSE48142	(8)
9.	SM003 - icSARS CoV, SARS MA15 wild type and SARS BatSRBD mutant virus infections of C57BL6 mice - A time course	C57BL6	icSARS CoV, Wild Type SARS MA15 or SARS BatSRBD mutant viruses	GSE50000	(8)
10.	The PDZ-binding motif of SARS-CoV envelope protein is a determinant of viral pathogenesis	BALB/c Ola Hsd mice	MA15	GSE52920	(10)
11.	Genome wide identification of SARS-CoV susceptibility loci using the collaborative cross	C57BL/6J	MA15	GSE64660	
12.	Mouse lung tissue transcriptome response to a mouse-adapted strain of SARS-CoV in wild type C57BL6/NJ mice and TLR3-/- mice	C57BL6/NJ	MA15	GSE68820	(11)

## Results

In total, 9,692 genes were differentially expressed genes (DEGs) between mock-infected and virally infected models in the 12 studies. Thirty-eight genes that were DEGs in at least 9 out of 12 studies (75%) were considered common DEGs due to SARS-COV infection of the lungs in the first 48 h post-infection. These genes are listed in Table 2.

**Table 2:** Genes symbols and description for the common DEGs in 9 out of the 12 transcriptomics datasets due to SARS-CoV infection of the lung in the first 48 h post-infection.

**Genes symbol**	**Description**	**Number of studies where DEG was identified**
DDX58	DExD/H-box helicase 58	11
IFI44	interferon induced protein 44	11
IFIT1	interferon induced protein with tetratricopeptide repeats 1	11
IFIT2	interferon induced protein with tetratricopeptide repeats 2	11
IFIT3	interferon induced protein with tetratricopeptide repeats 3	11
ISG15	ISG15 ubiquitin like modifier	11
MX1	MX dynamin like GTPase 1	11
MX2	MX dynamin like GTPase 2	11
OAS3	2′-5′-oligoadenylate synthetase 3	11
XAF1	XIAP associated factor 1	11
BST2	bone marrow stromal cell antigen 2	10
CXCL10	C-X-C motif chemokine ligand 10	10
DHX58	DExH-box helicase 58	10
IFIH1	interferon induced with helicase C domain 1	10
IL6	interleukin 6	10
IRF7	interferon regulatory factor 7	10
OAS2	2′-5′-oligoadenylate synthetase 2	10
PARP14	poly(ADP-ribose) polymerase family member 14	10
RSAD2	radical S-adenosyl methionine domain containing 2	10
SP100	SP100 nuclear antigen	10
STAT1	signal transducer and activator of transcription 1	10
USP18	ubiquitin specific peptidase 18	10
BATF2	basic leucine zipper ATF-like transcription factor 2	9
CXCL11	C-X-C motif chemokine ligand 11	9
EPSTI1	epithelial stromal interaction 1	9
HERC6	HECT and RLD domain containing E3 ubiquitin protein ligase family member 6	9
IFI35	interferon induced protein 35	9
IFITM3	interferon induced transmembrane protein 3	9
ISG20	interferon stimulated exonuclease gene 20	9
PARP9	poly(ADP-ribose) polymerase family member 9	9
PLAC8	placenta associated 8	9
RTP4	receptor transporter protein 4	9
SAMD9L	sterile alpha motif domain containing 9 like	9
SP110	SP110 nuclear body protein	9
TRAFD1	TRAF-type zinc finger domain containing 1	9
TRIM21	tripartite motif containing 21	9
ZBP1	Z-DNA binding protein 1	9
ZC3HAV1	zinc finger CCCH-type containing, antiviral 1	9

### Species-Specific Response to SARS-CoV Infection

**Figure 1:**
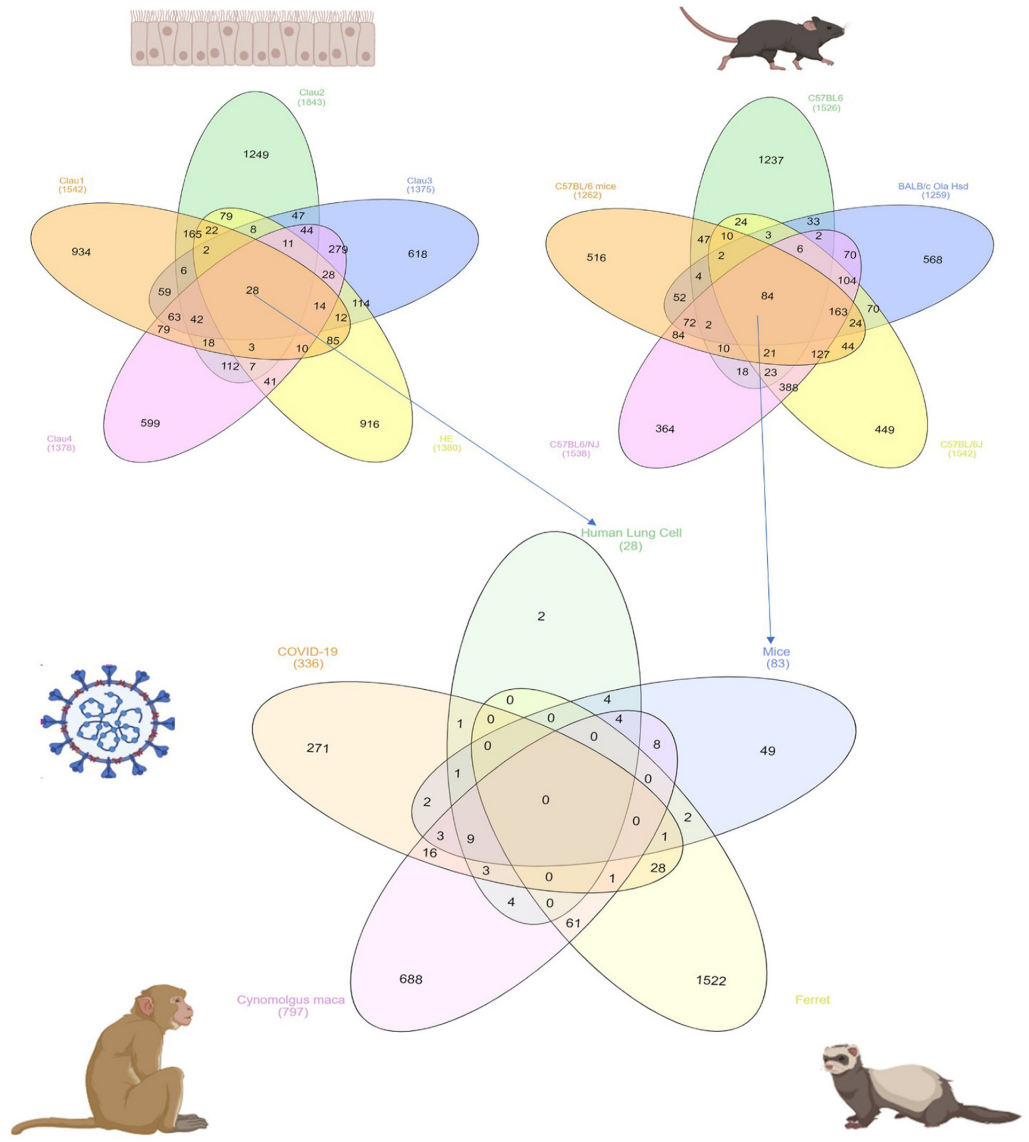
Common DEGs among mammals and human cells infected with SARS-CoV virus extracted from publicly available transcriptomics datasets.

In order to identify DEG in SARS-CoV infected lung tissue-specific to each of the models used and those which are shared, we intersected the DEGs from datasets that use the same model. Human epithelial cells datasets (GSE17400, GSE33267, GSE37827, GSE47960, and GSE48142), mice datasets (GSE33266, GSE50000, GSE52920, GSE64660, and GSE68820), Ferret (GSE11704) and Cynomolgus macaques (GSE23955) were all intersected with the COVID-19 infected epithelial cells dataset as shown in Figure 1. The number of DEG intersected between different species is listed in the Table 3. Epithelial cells infected with SARS-CoV-2 shared 9 DEGs (MX1, OAS3, XAF1, IFI44, MX2, IRF7, STAT1, IFIT3, and IFIT1) with Human Lung Cells, Mice, and Cynomolgus maca.

**Table 3:** Number of shared DEGs in different models infected with SARS-CoV extracted from publicly available transcriptomics datasets.

**Model infected**	**Number of DEGs**
[COVID-19]	271
[COVID-19] and [Cynomolgus maca]	16
[COVID-19] and [Ferret]	28
[COVID-19] and [Ferret] and [Cynomolgus maca]	1
[COVID-19] and [Human lung cell]	1
[COVID-19] and [Human lung cell] and [Cynomolgus maca]	3
[COVID-19] and [Human lung cell] and [Mice]	1
[COVID-19] and [Human lung cell] and [Mice] and [Cynomolgus maca]	9
[COVID-19] and [Mice]	2
[COVID-19] and [Mice] and [Cynomolgus maca]	3
[COVID-19] and [Mice] and [Ferret]	1
[Cynomolgus maca]	688
[Ferret]	1,522
[Ferret] and [Cynomolgus maca]	61
[Human lung cell]	2
[Human lung cell] and [Cynomolgus maca]	4
[Human lung cell] and [Mice]	4
[Human lung cell] and [Mice] and [Cynomolgus maca]	4
[Mice]	49
[Mice] and [Cynomolgus maca]	8
[Mice] and [Ferret]	2

### The Identified Genes Are Involved in the Immune Response Against RNA Viruses

**Figure 2:**
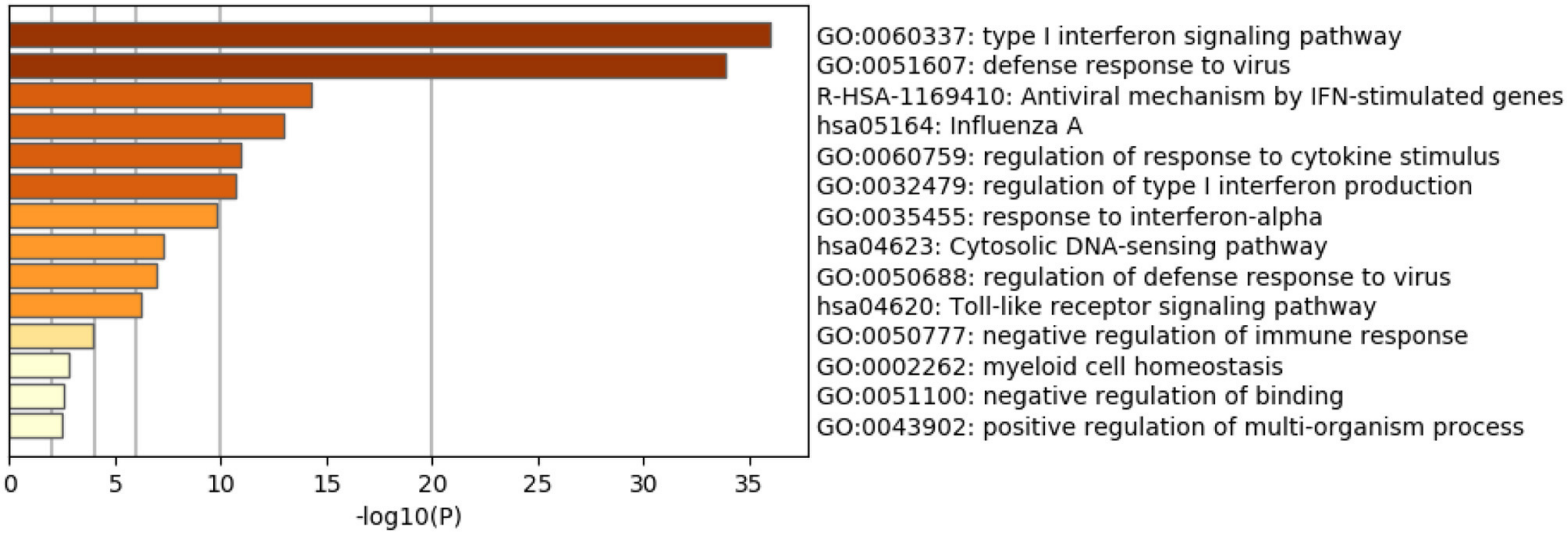
Enriched Ontology Clustering for the 38 identified DEGs.

As expected, the top genes identified are involved in innate immune responses against RNA viruses. These include the cytosolic DNA-sensing pathway, Toll-like receptor signaling pathway, and negative regulation of binding. Interferon (IFN) response to viral infections such as type I interferon signaling pathway, defense response to the virus, the antiviral mechanism by IFN-stimulated genes, regulation of type I interferon production, response to interferon-alpha, and regulation of defense response to virus and Influenza A, were also upregulated. Genes that play significant roles in activating immune systems such as regulation of response to cytokine stimulus, negative regulation of immune response, myeloid cell homeostasis, and positive regulation of the multi-organism process are also upregulated (Figure 2 and Table 4).

**Table 4:** Enriched Ontology Clustering for the 38 DEGs identified with Genes symbols in each category.

**Category**	**Term**	**Description**	**LogP**	**Log (*q*-value)**	**InTerm_InList**	**Symbols**
GO biological processes	GO:0060337	Type I interferon signaling pathway	−36.0121	−31.994	19/95	BST2, IFI35, IFIT2, IFIT1, IFIT3, IRF7, ISG20, MX1, MX2, OAS2, OAS3, SP100, STAT1, ISG15, IFITM3, USP18, XAF1, ZBP1, RSAD2, TRIM21, DDX58, IL6, CXCL10, CXCL11, PARP14, PARP9, ZC3HAV1, DHX58
GO biological processes	GO:0051607	Defense response to virus	−33.8766	−30.160	22/248	BST2, IFIT2, IFIT1, IFIT3, IL6, CXCL10, IRF7, ISG20, MX1, MX2, OAS2, OAS3, STAT1, ISG15, IFITM3, DDX58, ZC3HAV1, RTP4, IFIH1, DHX58, PARP9, RSAD2, IFI44, PLAC8, BATF2
Reactome gene sets	R-HSA-1169410	Antiviral mechanism by IFN-stimulated genes	−14.292	−11.274	9/81	IFIT1, MX1, MX2, OAS2, OAS3, STAT1, ISG15, USP18, DDX58
KEGG pathway	hsa05164	Influenza A	−12.9643	−9.967	10/173	IL6, CXCL10, IRF7, MX1, OAS2, OAS3, STAT1, DDX58, IFIH1, RSAD2, BST2, SP100, TRIM21, IFITM3, PARP14, PARP9, IFIT1
GO biological processes	GO:0060759	Regulation of response to cytokine stimulus	−10.9357	−8.032	9/188	IL6, IRF7, STAT1, USP18, DDX58, PARP14, IFIH1, ZBP1, PARP9, ZC3HAV1
GO biological processes	GO:0032479	Regulation of type I interferon production	−10.6988	−7.826	8/127	IRF7, TRIM21, STAT1, ISG15, DDX58, IFIH1, DHX58, ZBP1, TRAFD1, USP18, PARP14, PARP9, RSAD2, CXCL10, BST2, IL6, IFIT1, SP100, BATF2
GO biological processes	GO:0035455	Response to interferon-alpha	−9.83448	−7.021	5/21	BST2, IFIT2, IFIT3, MX2, IFITM3, STAT1, XAF1
KEGG pathway	hsa04623	Cytosolic DNA-sensing pathway	−7.31693	–.600	5/63	IL6, CXCL10, IRF7, DDX58, ZBP1, USP18, TRIM21
GO biological processes	GO:0050688	Regulation of defense response to virus	−6.93272	−4.247	5/75	IFIT1, STAT1, DDX58, DHX58, PARP9, BST2, IL6, RSAD2
KEGG pathway	hsa04620	Toll-like receptor signaling pathway	−6.22049	−3.626	5/104	IL6, CXCL10, IRF7, CXCL11, STAT1, OAS2, ISG15, IFI44, PLAC8, HERC6, DHX58, SP100
GO biological processes	GO:0050777	negative regulation of immune response	−3.98249	−1.608	4/156	BST2, TRAFD1, PARP14, DHX58
GO biological processes	GO:0002262	Myeloid cell homeostasis	−2.78923	−0.508	3/148	IL6, STAT1, ISG15, IRF7, BATF2
GO biological processes	GO:0051100	Negative regulation of binding	−2.61062	−0.352	3/171	IFIT2, IFIT1, SP100, PARP9
GO biological processes	GO:0043902	Positive regulation of multi-organism process	−2.50748	−0.264	3/186	IFIT1, TRIM21, DHX58, IFITM3

### IFITM3, OAS2, and MX1 Showed the Highest Upregulation in SARS-CoV-2 Infected Epithelial Cells

**Figure 3:**
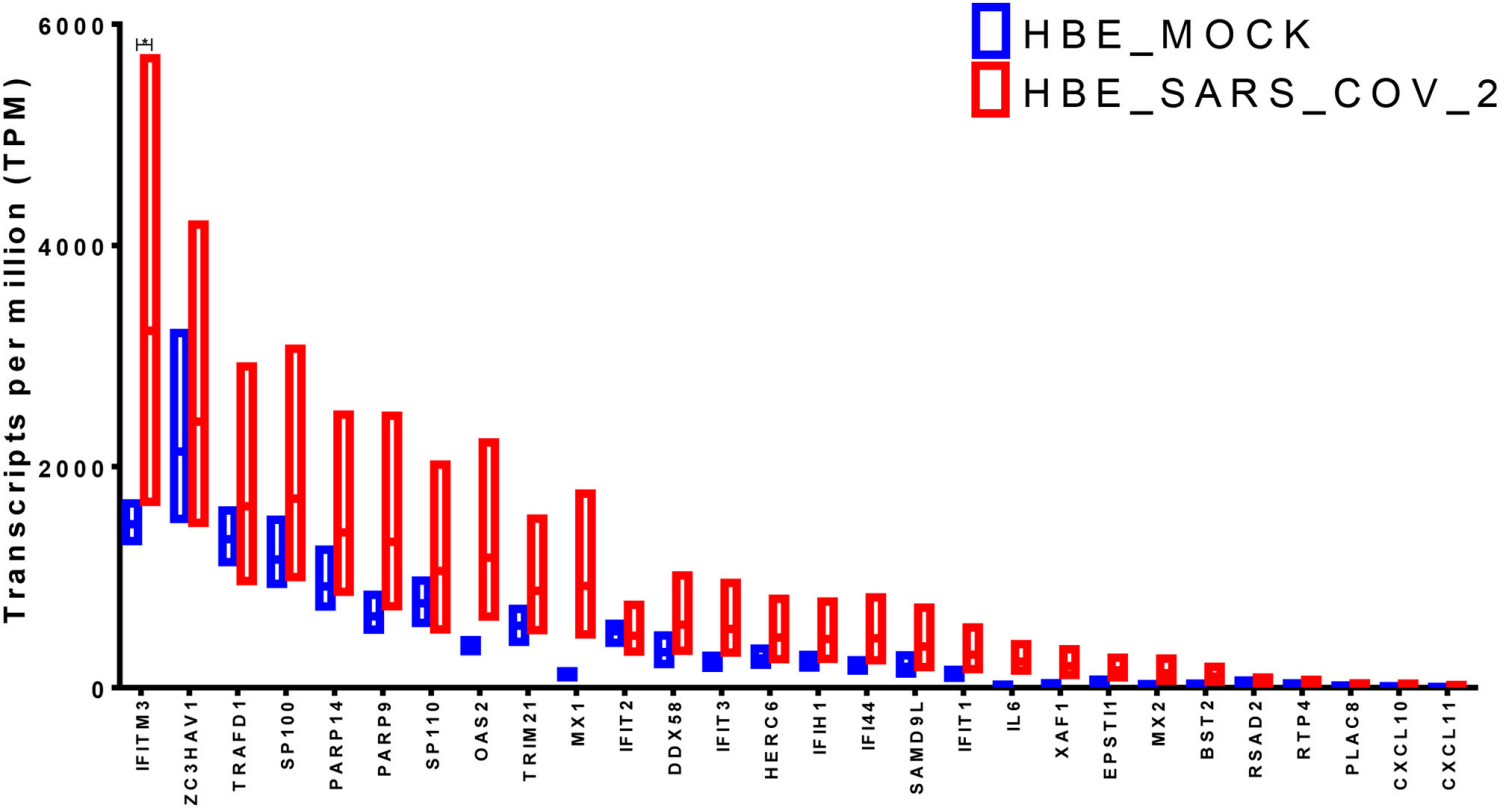
Gene expression of the shortlisted genes in healthy epithelium infected with SARS-CoV-2 compared to mock-infected cells from the transcriptomics dataset (GSE147507).

The identified genes expression levels were higher in human bronchial epithelium infected with SARS-CoV-2 compared to those mock-infected (Figure 3). However, only IFITM3 showed a significant difference (*p* < 0.05), while two other genes OAS2 and MX1 showed a trend of enhancement, although it was not statistically significant (*p* = 0.06 using the two-stage linear step-up procedure of Benjamini, Krieger, and Yekutieli). IFITM3 mRNA levels were one of the highly expressed genes compared to the other identified genes at baseline in mock-infected HBE and were further induced by the virus, which results in overall high mRNA levels.

### Valproic Acid Can Upregulate the IFITM3 mRNA Expression

Next, we searched the Comparative Toxicogenomics Database (http://ctdbase.org/) to identify drugs/chemicals that might affect the mRNA expression of IFITM3 in at least two reference studies (14). Interestingly valproic acid, carbon nanotubes, nickel, and tert-butylhydroperoxide were shown to upregulate IFITM3 expression while pirinixic acid, acetaminophen, and Ethinyl estradiol decreased such an expression (Table 5).

**Table 5:** Chemicals shown to upregulate or downregulate IFITM3 mRNA expression in at least two studies as shown in the Comparative Toxicogenomics Database (http://ctdbase.org/).

**Chemical name**	**Chemical ID**	**CAS RN**	**Interaction**	**Effect on IFITM3 mRNA expression**	**Reference count**	**Organism count**
Valproic acid	D014635	99-66-1	Valproic acid results in increased expression of IFITM3 mRNA	increases	3	2
Nanotubes, carbon	D037742		Nanotubes, carbon analog results in increased expression of IFITM3 mRNA	increases	2	1
Nickel	D009532	7440-02-0	Nickel results in increased expression of IFITM3 mRNA	increases	2	1
Tert-butylhydroperoxide	D020122	75-91-2	tert-Butylhydroperoxide results in increased expression of IFITM3 mRNA	increases	2	1
Pirinixic acid	C006253	50892-23-4	Pirinixic acid results in decreased expression of IFITM3 mRNA	decreases	3	2
Acetaminophen	D000082	103-90-2	Acetaminophen results in decreased expression of IFITM3 mRNA	decreases	2	1
Ethinyl estradiol	D004997	57-63-6	Ethinyl Estradiol results in decreased expression of IFITM3 mRNA	decreases	2	2

**Figure 4:**
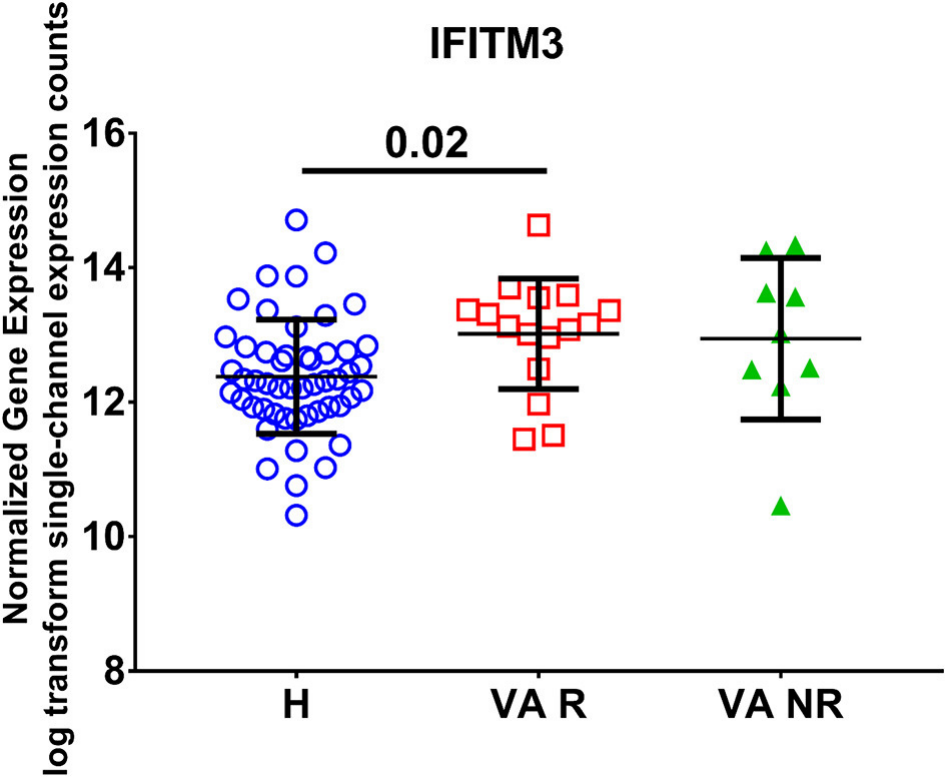
Normalized gene expression of IFITM3 in the blood of patients on responder valproic acid monotherapy (VA R) and non-response (VA NR) compared to healthy controls (H) extracted from the transcriptomic dataset (GSE143272).

In order to examine the effect of valproic acid therapy on the IFITM3 mRNA expression in immune cells of the blood, a publicly available transcriptomics dataset (GSE143272) was extracted and reanalyzed. Healthy controls were compared to responders and non-responders patients on valproic acid therapy. We found upregulation of the mRNA expression of IFITM3 in patients, and the difference was significant in the responder group only (*p* < 0.05) compared to healthy controls (Figure 4).

## Discussion

In response to viral RNAs, like in the case of SARS-CoV-2, the innate immune system will unleash interferon (IFN), to activate antiviral mechanisms and effector cells like natural killers (15). In mice infected with SARS-CoV, a delayed and prolonged type I interferon (IFN-I) signaling leads to lung immunopathology as it promotes the accumulation of pathogenic inflammatory cells with increased lung cytokine/chemokine levels and vascular leakage (16). This prolonged IFN-I and virally induced IL-10 set the scene for secondary bacterial infection, which can add a strong IL1β and TNFα-mediated inflammatory response to magnify lung damage (17). Understanding how SARS-CoV-2 can manipulate IFN is vital in deciphering the battle of the body against the viral spread and consequence.

Our reanalysis of transcriptomic data showed that although the IFN pathway is upregulated consistently in SARS-CoV related infection, SARS-CoV-2 showed specific upregulation of the gene for a unique interferon-induced protein, namely IFITM3. IFITM3 is a 15-kDa protein that localizes to endosomes and lysosomes and is possibly acquired by mammalian ancestral cells via horizontal gene transfer (18).

Interferon-induced transmembrane proteins (IFITMs 1, 2, and 3) are innate immune responders to virus infections as they regulate the fusion of invading virus and endocytic vesicles and direct it to the lysosomes (19, 20). IFITM3 can further alter membrane rigidity and curvature to inhibit virus membrane fusion (21). Such action is important to prevent the release of viral particles into the cytoplasm, which controls viral spread (22). During influenza A infection of human airway epithelial cells, IFITM3 was shown to clusters on virus-containing endosomes and lysosomes within few hours post-infection, indicating its role in the early phase of viral entry (23). Even platelets and megakaryocytes were shown to remarkably upregulate IFITM3 to prevent viral progression during influenza infection (24).

The epithelial cell and resident leukocytes in lung upper and lower airways that constitutively express IFITM3 can withstand viral infections, and this is vital to decide viral tropism as viruses favor cells with low IFITM3 expression (25). IFITM3 enhances the accumulation of CD8+ T cells in airways to promote mucosal immune cell persistence (26). Lung and circulating immune cells were reported to express less IFITM3 than other tissues, and this was a suggestive reason for COVID-19 severity and cytokine release syndrome (27).

Interestingly, IFITM3-rs12252-C/C SNP prevalence in the Chinese population is 26.5%, and recent research confirmed that SNPs in IFITM3 could change the severity of influenza infection, as was shown in one case with COVID-19 (28). IFITM3 polymorphisms have been linked with hospitalization and mortality during influenza virus infection (29).

Expressing the gene is not the only prerequisite to the antiviral action of IFITM3, as it was found recently that within the protein, an amphipathic helix is critical for its blocking effect of viral fusion of similar pathogenic viruses like influenza A virus and Zika virus (30). Another factor that regulates the IFITM3 trafficking specificity to such viruses is that it requires S-palmitoylation (19, 20). S-palmitoylation (S-PALM) is the reversible process of linking a fatty acid chain to cysteine residues of the substrate protein (31). Multiple zinc finger DHHC domain-containing palmitoyltransferases (ZDHHCs) can palmitoylate IFITM3 to make it a fully functional antiviral protein (32). It seems that bats (order *Chiroptera*), which act as natural hosts for many viral infections, use IFITM3 as an antiviral mechanism if there is S-palmitoylation of the protein; however, if this modification is disturbed, the bat can develop viral infection (33). Based on that, we can suggest that severe COVID-19 cases might be due to either non-functional IFITM3 by SNP, failure of lung cells to upregulate IFITM3 in response to interferon, a mutation in amphipathic helix sequence or modification in S-palmitoylation. Further examination and screening for the IFITM3 dynamics in COVID-19 might explain the possible therapeutic and diagnostic options.

Our toxicogenomic analysis showed that valproic acid increased the mRNA expression of IFITM3, supporting a new report that the SARS-CoV-2-human protein-protein interaction map showed that valproic acid might be a potential repurposing drug for COVID-19 (34). Virtual screening, docking, binding energy calculation, and simulation show that valproic acid forms stable interaction with nsP12 of CoV and can inhibit its function (14). Valproic acid is currently used for the treatment of epilepsy and known to target histone deacetylases (HDACs) that modify the gene expression epigenetically (35). Valproic acid was shown to inhibit mature and fully infectious enveloped viruses release as it alters cellular membrane composition (36). The modest and broad antiviral activity of valproic acid made the drug an attractive possibility due to its availability, and limited side effects for a short term of use during acute viral disease (37). The reported side effects like hepatotoxicity and teratogenicity are mainly associated with the parental compound valproate and can be avoided by the use of its derivatives like Valpromide (VPD) and valnoctamide (VCD). A recent open-label proof-of-concept trial of 10 days Intravenous Valproic Acid for Severe COVID-19 showed a 50% reduction in the case fatality rate and length of stay (38). More studies are needed to explore the promising potential of valproic acid in the treatment of COVID-19.

One limitation of the study is that it is based on the publicly available transcriptome dataset, which is limited in number, partly because this is a novel disease, but also because ongoing lockdowns have made it challenging for scientists to carry out the extensive laboratory work required.

## Conclusion

Our evaluation showed that the analysis of publicly available transcriptomic data could be a reasonable approach to identify the novel target and suggest drugs that can modify the action of such targets during the early phases of emerging infections like COVID-19 until a complete understanding of the disease become clear. This can justify the experimental use of clinically approved drugs and guide the clinicians in their limited options against such lethal disease.

## Data Availability Statement

All datasets presented in this study are included in the article/supplementary material.

## Author Contributions

All authors listed have made a substantial, direct and intellectual contribution to the work, and approved it for publication.

## Conflict of Interest

The authors declare that the research was conducted in the absence of any commercial or financial relationships that could be construed as a potential conflict of interest.
